# Predictive Efficacy of Delta Neutrophil Index in Diagnosis of Acute and Complicated Appendicitis

**DOI:** 10.7759/cureus.14748

**Published:** 2021-04-29

**Authors:** Birkan Birben, Gökhan Akkurt, Tezcan Akın, Aziz A Surel, Mesut Tez

**Affiliations:** 1 General Surgery, Ankara City Hospital, Ankara, TUR

**Keywords:** delta neutrophil index, acute appendicitis, complicated appendicitis

## Abstract

Background

The delta neutrophil index has been accepted as an inflammatory marker, especially in sepsis. This study aimed to evaluate the effectiveness of the delta neutrophil index in predicting acute and complicated appendicitis.

Methods

Patients aged 18 years and over who underwent appendectomy were reviewed. The demographic features, pathology results, and the delta neutrophil index, leukocyte, and C-reactive protein levels were evaluated. According to the pathology results, the patients were grouped as those having a normal appendix or acute appendicitis.

Results

In this study, 74 (8.1%) of the patients had a normal appendix, and 718 (86.1%) were diagnosed with simple appendicitis, and 116 (13.9%) with complicated appendicitis. In the acute appendicitis group, the leukocyte value and delta neutrophil index were found to be statistically significantly higher than in the normal appendix group. Age, C-reactive protein, and the delta neutrophil index ​​were statistically significantly higher in the complicated appendicitis group. In the receiver operating characteristic curve analysis for the prediction of acute appendicitis, the area under the curve values for leukocyte and the delta neutrophil index were calculated as 0.780 and 0.741, respectively. In predicting complicated appendicitis, the area under the curve of the delta neutrophil index and C-reactive protein were 0.671 and 0.709, respectively.

Conclusion

The delta neutrophil index was more significant than leukocyte values in diagnosing acute and complicated appendicitis. We consider that the delta neutrophil index ​​is an effective and reliable parameter in diagnosing acute appendicitis and differentiating simple/complicated appendicitis, especially when combined with the analysis of leukocyte and C-reactive protein.

## Introduction

Acute appendicitis is characterized by bacterial colonization due to the obstruction of the appendix lumen [1]. Appendicitis becomes complicated by an abscess, necrosis, or perforation of the appendix secondary to an infection [2]. The prolonged time from the onset of symptoms to the diagnosis increases the risk of appendiceal perforation [3]. C-reactive protein (CRP), leukocyte and bilirubin are useful biochemical markers in evaluating the severity of inflammation [4]. The delta neutrophil index (DNI) is a marker obtained by measuring the fraction of circulating immature granulocytes and increases in infection and inflammation states [5]. DNI is calculated using the following formula: DNI(%) = (neutrophil% + eosinophil%) - (polymorphoneutrophil%) [6]. In the current study, we aimed to evaluate the effectiveness of DNI%, which is widely used as an inflammatory marker, especially in sepsis, in predicting acute and complicated appendicitis.

## Materials and methods

Ethics committee approval was received from the local ethics committee. A total of 934 patients aged 18 years and over who underwent surgery with the diagnosis of acute appendicitis between April 2019 and March 2020 in the emergency surgery department of the hospital were retrospectively analyzed. The demographic features, pathology results, and DNI%, CRP, and leukocyte levels at the time of the first emergency presentation were evaluated. Patients with a history of immunosuppressive therapy, pregnant women, patients that underwent surgery for different reasons, those that underwent additional appendectomy or interval appendectomy, and those with malignant pathologies were excluded from the study. According to the pathology results, the patients were divided into two groups: those having a normal appendix, and those diagnosed with acute appendicitis. The acute appendicitis group was further divided into two more groups as simple and complicated, depending on the absence or presence of an abscess, necrosis, perforation, and generalized peritonitis.

Statistical analysis

The data were analyzed statistically using SPSS software v. 23.0 (IBM SPSS Statistics for Windows, IBM Corp, Armonk, NY). The Shapiro-Wilk test, skewness, and kurtosis values were used to analyze the data distribution. All data were expressed as mean ± standard deviation or median (interquartile range) according to data distribution. The statistical analysis of the results was performed using unpaired Student’s t-tests and ANOVA models (with Tamhane’s post-hoc test) for the normally distributed data. For the data without normal distribution, the Mann-Whitney U and Kruskal-Wallis tests were used. The association between the categorical variables was tested using the chi-square or Fisher’s exact test. Significance was considered when P < 0.05 and when supported by the Bonferroni multiple testing correction. 

The sample size was estimated with G*Power for Windows [7]. The goal was to detect a negative appendicectomy rate of 10%. Therefore, assuming a two-sided alpha 0.05, a sample size of at least 32 patients in each group to achieve 90% power was anticipated.

We measured the prognostic performance of the laboratory parameters using receiver operating characteristic (ROC) curves and calculated sensitivity, specificity, positive predictive value (PPV), negative predictive value (NPV), positive likelihood ratio (+LR), and negative likelihood ratio (−LR) for different cut-off values.

## Results

**Figure 1:**
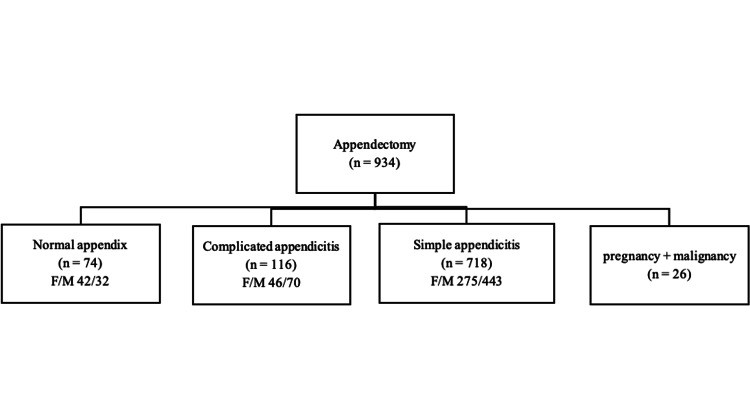
Distribution of groups F: Female, M: Male

Of the 934 patients who underwent appendectomy, 16 were excluded due to the detection of appendectomy malignancies in the pathological analysis and a further 10 were excluded since they had the operation during pregnancy. According to the pathology reports of the remaining 908 patients, 74 (8.1%) had a normal appendix, while 718 (86.1%) were diagnosed with simple appendicitis, and 116 (13.9%) with complicated appendicitis (Figure 1). The whole sample consisted of 363 (40%) women and 545 (60%) men, with the mean age being calculated as 36 ± 15 (18-87) years.

Of the patients in the normal appendix group, 42 (56.8%) were female and 32 (43.2%) were male. The mean age of this group was 38 ± 15 years. The mean leukocyte mean value was 9.864 ± 3.522 x 106/L, the median DNI% was -2.75[(-4.90)-(-.30)], and the median CRP was 5 (3-28) mg/dl. In the simple appendicitis group, there were 275 (38.3%) females and 443 (61.7%) males, with a mean age of 35 ± 14 years. In this group, the mean leukocyte value was 13.723 ± 4.379 x 106/L, the median DNI% was -.50 [(-2.10)-(1.00)], and the median CRP was 13 (3-49) mg/dl. Of the patients in the complicated appendicitis group, 46 (39.7%) were female and 70 (60.3%) were male, with the mean age being obtained as 42 ± 18 years. The mean leukocyte, median DNI%, and median CRP values were 14.357 ± 3.852 x 106/L, .75 [(-.75)-(2.5)], and 46 (14-120) mg/dl, respectively (Table 1).

**Table 1:** Comparison of the groups DNI; Delta neutrophil index, CRP; C-reactive protein, SD; Standard deviation *ANOVA test

		Normal appendix	Simple appendicitis	Complicated appendicitis	P* value
Age (years)	Mean	38	35	42	<0.005
	SD	15	14	18	
Leukocyte x10^6^/L	Mean	9.864	13.723	14.357	<0.005
	SD	3.522	4.379	3.852	
DNI%	Median	-2.75	-.50	.75	<0.005
	Percentile 25	-4.90	-2.10	-.75	
	Percentile 75	-.30	1.00	2.50	
CRP mg/dl	Median	5	13	46	<0.005
	Percentile 25	3	3	14	
	Percentile 75	28	49	120	

When the normal appendix and acute appendicitis groups were compared, there was no statistically significant difference in terms of age (p = .146). While the leukocyte and DNI% values ​​were significantly higher in the acute appendicitis group than in the normal appendix group (p = .000 and .001, respectively), there was no significant difference in the CRP values ​​of the two groups (p = .478). The age of the complicated appendicitis group was significantly higher than that of the simple appendicitis group (p = .000), but there was no significant difference in terms of leukocyte values (p = .142). The DNI% and CRP values ​​were significantly higher in the complicated appendicitis group compared to the simple appendicitis group (p = .002 and .000, respectively).

**Figure 2:**
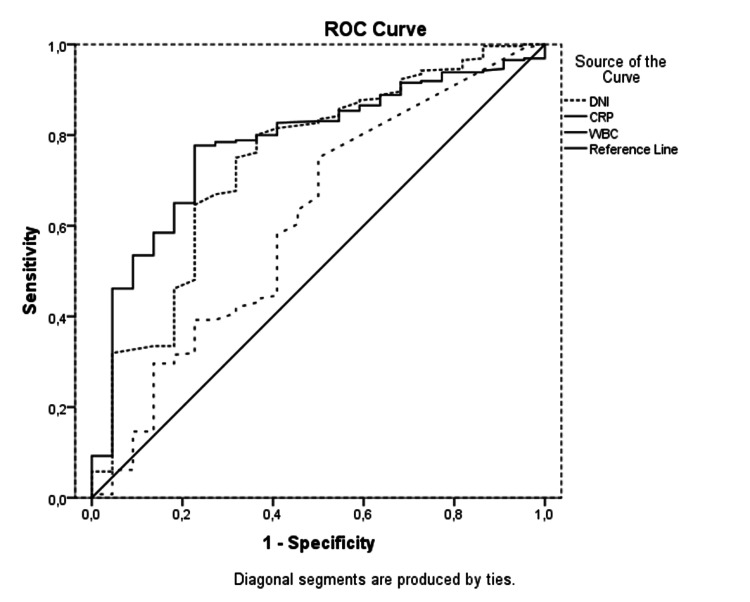
ROC analysis of acute appendicitis WBC; White blood cell, DNI; Delta neutrophil index, CRP; C-reactive protein

 In the ROC analysis for the prediction of acute appendicitis, the area under the curve (AUC) values of leucocyte, DNI%, and CRP were 0.780, 0.741, and 0.611, respectively (Figure 2). At the cut-off value of 11.245 x 106/L, the sensitivity, specificity, PPV and NPV of leukocyte were 73.38%, 72.97%, 96.84% and 19.57%, respectively. In predicting acute appendicitis, at the cut-off value of -1.05, DNI% had sensitivity, specificity, PPV and NPV 63.31%, 66.22%, 95.48% and 13.80%, respectively. For CRP, at the cut-off value 10.5 mg/dl, the sensitivity, specificity, PPV, and NPV were calculated as 58.08%, 59.09%, 94.38%, and 10.66%, respectively (Table 2).

**Table 2:** ROC analysis of acute appendicitis DNI; Delta neutrophil index, CRP; C-reactive protein, CI; confidence interval, AUC; area under the curve, PPV; positive predictive value, NPV; negative predictive value

	AUC	95% CI		Cut-off value	Sensitivity	Specificity	PPV	NPV
		Lower	Upper					
Leukocyte x10^6^/L	.780	.689	.871	11.245	73.38%	72.97%	96.84%	19.57%
CRP mg/dl	.611	.478	.744	10.5	58.08%	59.09%	94.38%	10.66%
DNI%	.741	.628	.854	-1.05	63.31%	66.22%	95.48%	13.80%

**Figure 3:**
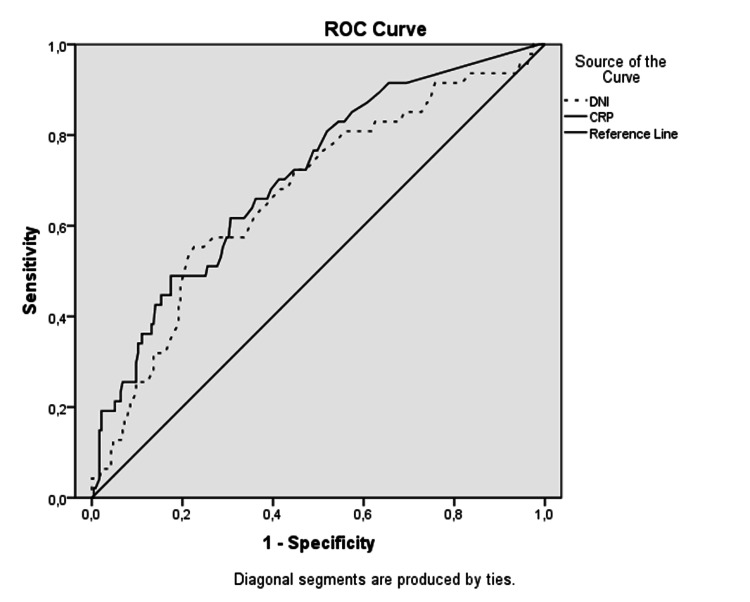
ROC analysis of complicated appendicitis DNI; Delta neutrophil index, CRP; C-reactive protein

The ROC analysis revealed that in predicting complicated appendicitis, the AUC values of leukocyte, DNI%, and CRP were 0.607, 0.671, and 0.709, respectively (Figure 3). At the cut-off value of 13.795 x106/L, leukocyte had a sensitivity of 54.31%, specificity of 15.22%, PPV of 51.11%, and NPV of 87.38%. When the cut-off value of DNI% was 0.25, this parameter had sensitivity, specificity, PPV, and NPV of 58.62%, 62.40%, 20.12%, and 90.32%. For CRP, at a cut-off value of 26 mg/dl, sensitivity, specificity, PPV, and NPV were calculated as 63.83%, 64.32%, 28.30%, and 88.96%, respectively (Table 3).

**Table 3:** ROC analysis of complicated appendicitis DNI; Delta neutrophil index, CRP; C-reactive protein, CI; confidence interval, AUC; area under the curve, PPV; positive predictive value, NPV; negative predictive value

	AUC	95% CI		Cut-off value	Sensitivity	Specificity	PPV	NPV
		Lower	Upper					
Leukocyte x10^6^/L	.607	.525	.690	13.795	54.31%	51.11%	15.22%	87.38%
CRP mg/dl	.709	.629	.789	26	63.83%	64.32%	28.30%	88.96%
DNI%	.671	.585	.757	.25	58.62%	62.40%	20.12%	90.32%

## Discussion

In a study conducted with 650 patients, Shin et al. argued that DNI% could be used as an effective marker in predicting acute appendicitis and differentiating simple/complicated appendicitis. In the same study, in the prediction of acute appendicitis, the AUC and cut-off values of DNI% were determined as 0.709 and 0.2, respectively, and the sensitivity and specificity of this parameter were 59.8% and 77.1%, respectively according to the ROC analysis. In predicting complicated appendicitis, DNI% had an AUC of 0.727, cut-off value of 0.6, sensitivity of 65%, and specificity of 71% [5]. In a study undertaken by Ünal, 438 patients were evaluated and DNI% was suggested as a marker that could be used in both predicting acute appendicitis and distinguishing complicated appendicitis. In the ROC analysis for the prediction of acute appendicitis, the AUC of DNI% was reported as 0.739 and the cut-off value was 0.4, at which the sensitivity and specificity were calculated to be 47% and 88.4%, respectively, while for the prediction of complicated appendicitis, these values were 0.979, 0.6, 94.4%, and 97.9%, respectively [8]. In another study by Shin et al., it was stated that among the elderly, DNI% was statistically significantly higher in those with perforated appendicitis than in non-perforated cases [9]. In the current study with 908 patients, compared to the patients with a normal appendix, we observed a higher DNI% in acute appendicitis (sensitivity 63.31%, specificity 66.22%), and when we compared the acute and complicated appendicitis groups, we determined a higher DNI% in the latter (sensitivity 58.62%, specificity 62.40%). However, the comparison of patients with perforated appendicitis and those with acute appendicitis did not reveal any significant difference. Thus, the effectiveness of DNI% in predicting acute and complicated appendicitis in our study was in agreement with the literature.

In a previous study, it was found that the leukocyte value was higher in patients with acute appendicitis than in those with a normal appendix, but they did not significantly when compared to the complicated appendicitis group [5]. In another study, it was advocated that the leukocyte value was significantly elevated in both acute and complicated appendicitis groups [8]. In contrast, Akai et al. showed that leukocyte did not provide significant results in the distinction of simple and complicated appendicitis [10]. In our study, we observed that the leukocyte values ​​were higher in patients with acute appendicitis than in those with a normal appendix, but we did not find a significant difference between simple and complicated appendicitis groups.

Shin et al. reported that the mean age was higher ​​in acute appendicitis than in patients with normal appendix, and in complicated appendicitis compared to simple appendicitis [5]. Similarly, other researchers also found the mean age to be high in the complicated appendicitis group compared to the simple appendicitis group [8, 11]. In our study, we determined that the mean age was not significant in the distinction between a normal appendix and acute appendicitis, but it was higher in the complicated appendicitis group compared to the other groups. The majority of patients with an advanced age presenting with the suspicion of acute appendicitis are diagnosed with complicated appendicitis due to inappropiate inflammatory response [12]. This theory is supported by our complicated appendicitis group mostly comprising older patients.

Shin et al. reported that the CRP value in acute appendicitis was higher than in patients with a normal appendix, but they did not detect any statistically significant difference between the two groups. However, in complicated appendicitis, the CRP value was found to be higher than in simple appendicitis cases [5]. Avanesov et al. and Kim et al. observed that the CRP values ​​of the patients with complicated appendicitis were statistically significantly higher than those with simple appendicitis [11, 13]. In our study, although the CRP value was higher in acute appendicitis than in patients with a normal appendix, there was no statistically significant difference. In addition, the CRP values ​​were statistically significantly higher in the complicated appendicitis group compared to the simple appendicitis group. Since the serum CRP level reaches the maximum level in 48 hours, we consider that elevated CRP can be very useful in the diagnosis of cases with a delayed diagnosis of appendicitis and complicated appendicitis.

Although there are many studies on DNI% in the literature, only a limited amount of research has been conducted to evaluate acute, simple, and complicated appendicitis together. Considering the appendicitis studies related to DNI%, the number of cases in our study was higher than those of previous studies. We can attribute this result to our hospital being a reference healthcare center in the region, and there is an influx of patients with appendicitis, including complicated cases referred from peripheral centers to our hospital. The limitation of this study is that it is a single-center study.

## Conclusions

The delta neutrophil index was more significant than leukocyte in diagnosing acute and complicated appendicitis. DNI% alone, or in combination with other parameters such as leukocyte and CRP, is an effective parameter in predicting acute appendicitis and distinguishing between simple/complicated appendicitis.

## References

[1] Yeşiltaş M, Karakaş DÖ, Gökçek B, Hot S, Eğin S (2018). Can Alvarado and Appendicitis Inflammatory Response scores evaluate the severity of acute appendicitis?. Ulus Travma Acil Cerrahi Derg.

[2] Eddama M, Fragkos KC, Renshaw S (2019). Logistic regression model to predict acute uncomplicated and complicated appendicitis. Ann R Coll Surg Engl.

[3] Papandria D, Goldstein SD, Rhee D (2013). Risk of perforation increases with delay in recognition and surgery for acute appendicitis. J Surg Res.

[4] Emmanuel A, Murchan P, Wilson I, Balfe P (2011). The value of hyperbilirubinaemia in the diagnosis of acute appendicitis. Ann R Coll Surg Engl.

[5] Shin DH, Cho YS, Cho GC (2017). Delta neutrophil index as an early predictor of acute appendicitis and acute complicated appendicitis in adults. World J Emerg Surg.

[6] Kim H, Kim Y, Lee HK, Kim KH, Yeo CD (2014). Comparison of the delta neutrophil index with procalcitonin and C-reactive protein in sepsis. Clin Lab.

[7] Faul F, Erdfelder E, Lang AG, Buchner A (2007). G*Power 3: a flexible statistical power analysis program for the social, behavioral, and biomedical sciences. Behav Res Methods.

[8] Ünal Y (2018). A new and early marker in the diagnosis of acute complicated appendicitis: immature granulocytes. Ulus Travma Acil Cerrahi Derg.

[9] Shin DH, Cho YS, Kim YS (2018). Delta neutrophil index: A reliable marker to differentiate perforated appendicitis from non-perforated appendicitis in the elderly. J Clin Lab Anal.

[10] Akai M, Iwakawa K, Yasui Y (2019). Hyperbilirubinemia as a predictor of severity of acute appendicitis. J Int Med Res.

[11] Avanesov M, Wiese NJ, Karul M (2018). Diagnostic prediction of complicated appendicitis by combined clinical and radiological appendicitis severity index (APSI). Eur Radiol.

[12] Garba S, Ahmed A (2012). Appendicitis in the elderly. Appendicitis - A Collection of Essays from Around the World.

[13] Kim M, Kim SJ, Cho HJ (2016). International normalized ratio and serum C-reactive protein are feasible markers to predict complicated appendicitis. World J Emerg Surg.

